# Cordyceps cicadae induces G2/M cell cycle arrest in MHCC97H human hepatocellular carcinoma cells: a proteomic study

**DOI:** 10.1186/1749-8546-9-15

**Published:** 2014-05-08

**Authors:** Hualin Wang, Jing Zhang, Wai-Hung Sit, Chung-Yung Jetty Lee, Jennifer Man-Fan Wan

**Affiliations:** 1 Food and Nutrition Division, School of Biological Sciences, The University of Hong Kong, Pokfulam Road, Hong Kong, SAR, China; 2 School of Biology and Pharmaceutical Engineering, Wuhan Polytechnic University, Wuhan, Hubei, China

## Abstract

**Background:**

*Cordyceps cicadae* is a medicinal fungus that is often used for treating cancer. However, the anticancer mechanisms of *C. cicadae* are largely unknown. This study aims to investigate the anticancer mechanisms of *C. cicadae* against hepatocellular carcinoma cells *in vitro* using a proteomic approach.

**Methods:**

Human hepatocellular carcinoma MHCC97H cells were treated with a water extract of *C. cicadae* (0, 100, 250, 500, and 1000 μg/mL) for 48 h and harvested for cell viability assays. The significant differences in protein expression between control and *C. cicadae*-treated cells were analyzed by two-dimensional gel-based proteomics coupled with matrix-assisted laser desorption ionization-time of flight mass spectrometry. Flow cytometry analysis was employed to investigate the cell cycle and cell death. The anticancer molecular mechanism was analyzed by whole proteome mapping.

**Results:**

The water extract of *C. cicadae* (0, 100, 250, 500, and 1000 μg/mL) inhibited the growth of MHCC97H cells in a dose-dependent manner *via* G2/M phase cell cycle arrest with no evidence of apoptosis. Among the identified proteins with upregulated expression were dynactin subunit 2, N-myc downstream-regulated gene 1, heat shock protein beta-1, alpha-enolase isoform 1, phosphatidylinositol transfer protein, and WD repeat-containing protein 1. Meanwhile, the proteins with downregulated expression were 14-3-3 gamma, BUB3, microtubule-associated protein RP/EB family member 1, thioredoxin-like protein, chloride intracellular channel protein 1, ectonucleoside triphosphate diphosphohydrolase 5, xaa-Pro dipeptidase, enoyl-CoA delta isomerase 1, protein-disulfide isomerase-related chaperone Erp29, hnRNP 2H9B, peroxiredoxin 1, WD-40 repeat protein, and serine/threonine kinase receptor-associated protein.

**Conclusion:**

The water extract of *C. cicadae* reduced the growth of human hepatocellular carcinoma MHCC97H cells *via* G2/M cell cycle arrest.

## Background

Primary liver cancer accounted for 6% of the total cancer cases worldwide in 2008 [1]. The highest incidences of liver cancer were in East Asia (Japan, Korea, and China) [2,3]. In China, liver cancer has the third highest estimated age-standardized cancer incidence rate in men and the fourth in women, and the second and third highest cancer mortality rates in men and women, respectively [1]. The high incidence of liver cancer in China is attributed to consumption of aflatoxin-contaminated grains, liver virus infection, and alcohol drinking [3]. Hepatitis B vaccination can effectively prevent liver cancer, but the treatment of liver cancer is still difficult [3,4].

Chinese medicine (CM) has been widely used in conjunction with chemotherapeutic drugs for liver cancer treatment in China with positive outcomes [5]. *Cordyceps* is a genus of ascomycete fungi belonging to the Clavicipitaceae family. All *Cordyceps* species are endo-parasitoids, and most of them parasitize insects and other arthropods. The *Cordyceps* genus includes nearly 400 species, and some of them have potential anticancer effects. *Cordyceps sinensis* is a medicinal fungus that has been used for cancer treatment in CM and Traditional Tibetan medicine since the 15th century [6,7]. The anticancer properties of *C. sinensis via* cancer cell apoptosis induction, proliferation inhibition, or both in various types of cancers, including leukemia, melanoma, Leydig tumor, breast cancer, and human hepatocellular carcinoma (HCC) have been investigated [8-10]. *C. sinensis* inhibited tumor metastasis *in vivo*[11,12]. Another well-known species, *Cordyceps militaris*, showed anticancer effects on leukemia, lung cancer, and breast cancer *in vitro* and *in vivo*[13-17], and its bioactive compound, cordycepin, exhibited cytotoxic and reactive oxygen species-generating activity in relation to cancer proliferation inhibition [18-22].

*Cordyceps cicadae*, another well-known medicinal mushroom that grows on larvae of *Cicadae flammata*, has been used in CM in China for the prevention and treatment of various diseases, including kidney disease, immune disease, and cancer [23-25]. *C. cicadae* exhibited immunoregulatory effects on human T lymphocytes and modulated the growth of mononuclear cells [26-29]. *C. cicadae* inhibited the growth of lung adenocarcinoma and melanoma *in vivo* and *in vitro*[30,31]. However, the anticancer mechanism of *C. cicadae* for liver cancer is still unknown.

In the last decade, proteomics has been widely used in medical studies for clinical biomarker identification, pathogenesis investigation, new drug discovery, pharmacological research, toxicological examination, and so on [32]. Most biological functions are transmitted via proteins such as enzymes, receptors, and structural components. Therefore, comprehensive proteomic analyses help us to understand the molecular modifications in physiological conditions [33]. This study aims to investigate the anticancer mechanisms of *C. cicadae* against HCC *in vitro* by two-dimensional gel-based proteomics coupled with matrix-assisted laser desorption ionization-time of flight (MALDI-TOF/TOF) mass spectrometry (MS), flow cytometry analysis, and proteome mapping.

## Materials and methods

### Cell culture and reagents

The MHCC97H cell line was purchased from the Liver Cancer Institute of Fudan University (China). MHCC97H cells were cultured in DMEM (Gibco BRL, USA) supplemented with 10% fetal bovine serum (Gibco BRL) in a humidified incubator containing 5% CO_2_ in air at 37°C, and subcultured with 0.25% trypsin-0.02% EDTA (Gibco BRL). A lyophilized hot water extract of wild-type *C. cicadae* (BioAsia Co., China) was dissolved in phosphate-buffered saline (PBS) and adjusted to a final concentration of 10 mg/mL.

### Cell proliferation assay

The dose-dependent effect of *C. cicadae* on cell viability was assessed by the MTT assay. Briefly, suspended MHCC97H cells (1 × 10^5^ cells/mL; 100 μL) were dispersed into the wells of 96-well microtiter plates. After 24 h of incubation, various concentrations of *C. cicadae* were added to each well and incubated for 48 or 72 h. Next, 10 μL of MTT solution (5 mg/mL 3-(4,5-dimethylthiazol-2-yl)-2,5-diphenyltetrazolium bromide dissolved in c-DMEM) (USB Corporation, USA) was added to each well, and incubated for 3 h at 37°C. The MTT solution was then removed and the insoluble purple formazan crystals formed were dissolved in 50 μL of isopropanol in 0.1 M HCl (MERCK, Germany). The optical density (OD) of each well was measured using a Bio-Rad 550 Microplate Reader (Bio-Rad, USA) at 595 nm with a reference wavelength of 655 nm. The percentage of cell viability was expressed as (A_treatment_ / A_control_) × 100%.

### Cell cycle analysis

The dose-dependent effect of *C. cicadae* on the cell cycle distribution was assessed by flow cytometry as described in our previous report [34]. Briefly, MHCC97H cells (1 × 10^5^ cells/mL) were treated with various concentrations of *C. cicadae* (0, 100, 250, 500, and 1000 μg/mL) for 48 h, and the cells were then harvested, fixed in 70% ethanol (MERCK, Germany), and stored at −20°C for 24 h until further analysis. Next, the cells were washed twice with ice-cold PBS, and incubated with RNase and propidium iodide (PI) (Sigma-Aldrich, USA) for 30 min. The PI-stained cells were excited at a wavelength of 488 nm and emitted at a maximum wavelength of 617 nm. Acquisition of 10,000 events was chosen for measurement of the DNA cell cycle distribution using a COULTER XL Flow Cytometer (Beckman and Coulter, USA). The distribution of cells in the different cell cycle phases shown in the DNA histograms was analyzed using Becton Dickinson Cell Fit Software (BD, USA).

### Sample preparation for proteomic analysis

MHCC97H cells were seeded in 100-mm culture dishes at 1 × 10^6^ cells/dish (1 × 10^5^ cells/mL), incubated overnight, and then treated with or without 500 μg/mL *C. cicadae* for 48 h. The cells were harvested by trypsinization, washed three times with PBS, and centrifuged in a Beckman Spinchron DLX (Beckman and Coulter) at 400 × *g* for 5 min. The cell pellet was resuspended in lysis buffer [1% Triton X-100 (USB Corporation), 25 mmol/L 4-(2-hydroxyethyl)-1-piperazineethanesulfonic acid (HEPES; Sigma-Aldrich), 150 mmol/L NaCl (UNIVAR, USA), 1 mmol/L EDTA disodium salt (Sigma-Aldrich), 1 mmol/L dithiothreitol (DTT; USB Corporation)] with Protease Inhibitor Cocktail Set III (AEBSF, aprotinin, bestatin, E-64, leupeptin hemisulfate, pepstatin A; Bio-Rad). The superfluous salt in the extract was removed by incubation with trichloroacetic acid (TCA)-acetone solution [20% TCA (MERCK, Germany), 20 mmol/L DTT in acetone (MERCK, Germany)] for 4 h at −40°C. The protein pellet was obtained by centrifugation at 15,800 × *g* for 30 min at 4°C. Excess TCA was removed by three washes with acetone containing 20 mmol/L DTT. The air-dried protein pellet was resuspended in buffer [7 mol/L urea (USB Corporation), 2 mol/L thiourea (Sigma-Aldrich), 4% 3-[(3-cholamidopropyl)dimethylammonio]-1-propanesulfonate (CHAPS; Sigma-Aldrich), 100 mmol/L DTT, 5% glycerol (USB Corporation)], and the protein solution was stored at −80°C until 2-DE analysis. The protein concentration was determined by the Bradford assay (Bio-Rad).

### Two-dimensional electrofocusing and polyacrylamide gel electrophoresis

The 2-DE procedures were carried as described in our previous report with modifications [35]. The samples were examined in duplicate and 12 gels (6 for control cells and 6 for *C. cicadae*-treated cells) were used in total. For the first-dimensional electrophoresis of proteins, protein samples (150 μg) were mixed with 350 μL of rehydration buffer [9.5 mol/L urea, 2% CHAPS, 0.28% DTT, 1% immobilized pH gradient buffer with pH 3–10 (Bio-Rad), 0.002% bromophenol blue (Sigma-Aldrich)] and then rehydrated for 10 h before isoelectric focusing *via* the following program: (a) linear increase up to 500 V in 1 h; (b) held at 500 V for 2 h; (c) linear increase up to 10,000 V in 4 h; (d) linear increase up to 10,000 V in 3 h; (e) held at 10,000 V to reach a total of 90,000 V × h. Focused immobilized pH gradient (IPG) gel strips were equilibrated for 15 min in a solution [50 mmol/L Tris–HCl, pH 8.8 (Bio-Rad), 6 mol/L urea, 30% glycerol, 2% sodium dodecyl sulfate (SDS; USB Corporation), containing 20 mmol/L DTT], followed by incubation with the same buffer containing 20 mmol/L iodoacetamide (Sigma-Aldrich) for another 15 min. The second-dimensional separation was performed in 1-mm-thick 12.5% polyacrylamide gels by sodium dodecyl sulfate-polyacrylamide gel electrophoresis (SDS-PAGE) at 15-mA current for 30 min followed by 30-mA current for the rest of the analysis.

### Image acquisition and analysis

After the electrophoresis, the gels were stained with SYPRORuby Protein Stain (Bio-Rad), scanned with a Molecular Imager PharosFX Plus System (Bio-Rad), and analyzed using PDQuest 8.0 (Bio-Rad) according to the following procedures: background subtraction, spot detection, and spot matching. The expression levels, expressed as the percentage volume (% vol), were exported. The relative intensities of spots were used for comparisons between the two groups and only those spots with a significant difference (>1.5-fold increase or decrease, *P* < 0.05) were selected for protein identification.

### Protein identification

Spots with differential expression (*P* < 0.05) after 500 μg/mL *C. cicadae* treatment were sent to the Genome Research Centre (The University of Hong Kong, Hong Kong) for protein identification by MALDI-TOF/TOF MS (4800 MALDI TOF/TOF Analyzer; Applied Biosystems, USA) analysis after trypsin digestion. The identification was performed by Mascot peptide mass fingerprinting, which can identify proteins from the NCBInr database with taxonomy limited to *Homo sapiens*. Mascot reported the molecular weight search (MOWSE) score, which was calculated as −10 × log_10_ (*P*), where *P* is the probability that the observed match is a random event. *P* was limited by the size of the sequence database being searched (limited by taxonomy), with conditions such as peptide fixed modification and variable modification and the settings for trypsin digestion. Each calculated value that fell within a given mass tolerance of an experimental value counted as a match. The threshold was accepted if an event occurred at random with a frequency of < 5%. In this study, a protein match with a score of > 71 was regarded as significant.

### Western blot analysis

Western blot analysis was performed with Mighty Small II SE250/260 Cell (Hoefer, USA) and TE 77 PWR Transfer (GE Healthcare, USA) electrophoresis units. Briefly, MHCC97H cells treated with or without various concentrations of *C. cicadae* (0, 100, 250, and 500 μg/mL) for 48 h were harvested, washed twice with PBS, and lysed in the lysis buffer for sample preparation described above. The lysates were centrifuged at 11000 × *g* for 30 min. The total protein concentration in the supernatant was determined by the Bradford assay (Bio-Rad). For immunoblotting, each protein extract was mixed with sample buffer [62.5 mmol/L Tris, pH 6.8 (Bio-Rad), 25% glycerol, 2% SDS, 350 mmol/L DTT, 0.01% bromophenol blue] at a ratio of 1:1 (v/v) and then kept in boiling water for 5 min. An aliquot (30 mg protein) of each sample was applied for electrophoresis in 12.5% SDS-PAGE gels with constant voltage (120 V) and transferred to a polyvinylidene difluoride membrane (GE Healthcare). The membranes were blocked with 5% skimmed milk in PBST [PBS, pH 7.4, 0.1% Tween-20 (USB Corporation)] for 3 h at room temperature, followed by incubation for 2 h with the following primary antibodies (ABCAM, UK): anti-CDK1 (ab32384; 1:2000); anti-cyclin B1 (ab32053; 1:2000); anti-HSP27 (ab2790; 1:2000); anti-PRDX1 (ab16805; 1:2000); anti-STRAP (ab46784; 1:2000). The membranes were washed three times with PBST, and incubated with HRP-conjugated goat anti-rabbit or anti-mouse IgG (Bio-Rad; 1:10 000) for 1 h at 37°C. After three washes with PBST, the membranes were developed using Inmun-Star HRP peroxide buffer (Bio-Rad). The relative molecular weight of each protein band was estimated using molecular markers (Precision Plus Protein Standards Dual Color; Bio-Rad). Each sample was measured three times.

### Statistical analysis

In cell proliferation assay, experiments were repeated three times, measurements within an experiment were done in six duplicate, and in cell cycle analysis, experiments were repeated three times. Data from three independent experiments were expressed as mean ± standard deviation (SD). The significance of differences in data was determined by Kruskal-Wallis one-way analysis of variance (ANOVA) followed by Dunnets *post hoc* test for determination of dose–response dependence. In proteomic analysis, six individual samples were prepared and run for 2D gel in each group (*i.e*., n = 6) the significance of differences in data was determined by a two-tailed Student’s *t*-test with Levene’s test for equality of variances. All analyses were performed using SPSS 17.0 (IBM, USA) with *P* < 0.05 as the significance level.

## Results

### Effects of *C. cicadae* on MHCC97H cell proliferation

**Figure 1:**
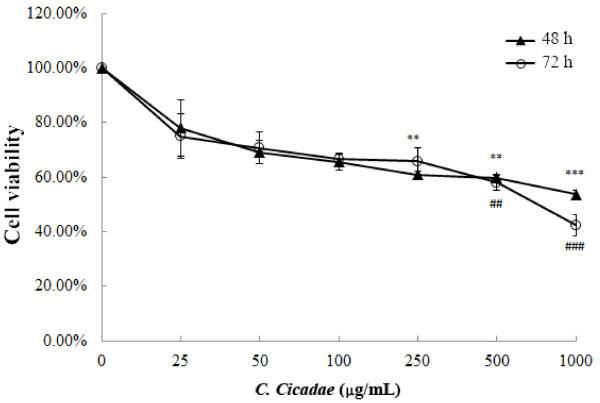
**The effects of *C*. *cicadae *on MHCC97H cell viability.** The cell viability was tested by MTT assay. Data were expressed as mean ± SD (n = 3 separate experiments). **,***, *P* < 0.01, 0.001 *vs*. control in 48 h treatment group, and ##, ###, *P* < 0.01, 0.001 *vs*. control in 72 h treatment group, respectively.

MHCC97H cells were grown in medium containing various concentrations of *C. cicadae* (0, 100, 250, 500, and 1000 μg/mL) for 48 or 72 h to investigate the effects of *C. cicadae* on cell proliferation. Dose-dependent inhibition of cell growth was observed with treatment of *C. cicadae* (*P* < 0.05) (Figure 1).

### Effect of *C. cicadae* on the cell cycle distribution of MHCC97H cells

**Figure 2:**
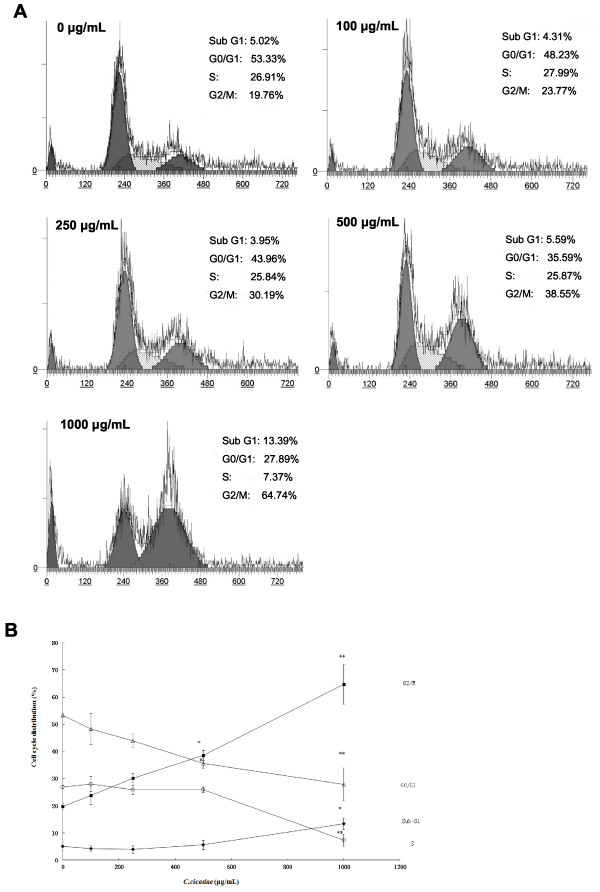
**The effects of *C*. *cicadae *on MHCC97H cell cycle. (A)** Cellular DNA contents in *C. cicadae*-treated and control cells were monitored by flow cytometry. The cell cycle distribution of sub-G1, G1, S and G2/M phase cells are presented as DNA histogram. **(B)** The percentage of cells in each cell cycle phase of MHCC97H after *C. cicadae* treatment were shown. The data were expressed as mean ± SD and were representative of three independent experiments. *, **, *P* < 0.05, 0.01, *vs*. control, respectively.

Figure 2A shows the effects of *C. cicadae* (0, 100, 250, 500, and 1000 μg/mL) treatment for 48 h on the cell cycle phase distribution of MHCC97H cells. As shown in Figure 2B, treatment with *C. cicadae* (100, 250, and 500 μg/mL) for 48 h resulted in cell accumulation in G2/M phase at 23.8%, 30.2%, and 38.6%, respectively, compared with the control cells (19.8%), and decreased the cell percentages in G0/G1 phase by 48.2%, 43.9%, and 35.6%, respectively, compared with the control cells (53.3%). Treatment with *C. cicadae* at 100, 250, and 500 μg/mL for 48 h did not affect the cell population in S phase (27.9%, 25.8%, and 25.9%, respectively, compared with the control cells (26.91%). Furthermore, most cells (64.74%) were arrested in G2/M phase with the highest dose of 1000 μg/mL *C. cicadae*. The sub-G1 apoptotic fraction of cells showed no significant changes when *C. cicadae* (100–500 μg/mL) was added, suggesting that no apoptosis occurred during the period of examination.

**Figure 3:**
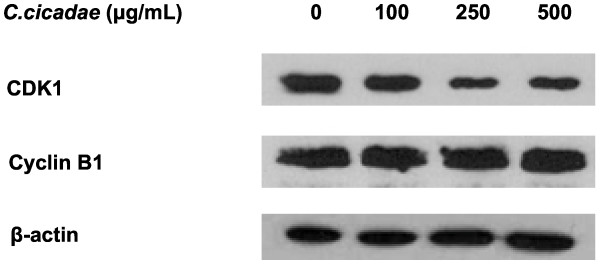
**The effects of *C*. *cicadae *on cell cycle checkpoint related proteins.***C. cicadae* treatment decreased the expression of CDK1 in MHCC97H cells.

CDK1/cyclin B complex is involved in the G2/M phase checkpoint by regulating the process of M phase [36]. Figure 3 shows that while the CDK1 protein level was decreased in MHCC97H cells after *C. cicadae* treatment, the cyclin B1 protein level was unaffected.

### Differential expression of proteins in *C. cicadae*-treated MHCC97H cells

**Figure 4:**
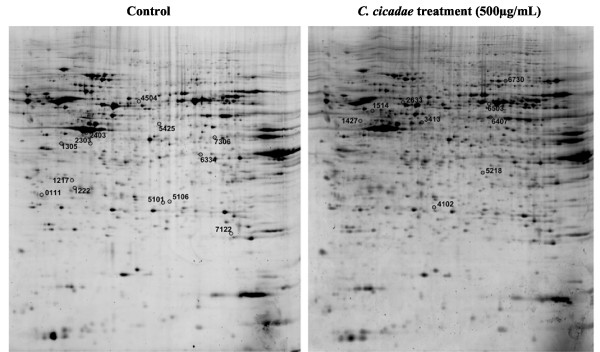
**Representative of 2-DE gel proteomic maps of MHCC97H cells with (left) or without (right) *C*. *cicadae *treatment.** The labelled spots were representative of proteins with significant change.

2-DE analyses were performed for each treated sample and repeated six times. Figure 4 shows representative gel images in which more than 1000 spots were detected on each gel. Proteins within the range of 15–225 kDa and having isoelectric points between 3 and 10 were well separated. Table 1 presents the differential expression levels of the identified proteins in MHCC97H cells without (control) and with *C. cicadae* treatment. Twenty-eight proteins with significant (*P* < 0.05) changes of > 1.5-fold in volume intensity were selected and further analyzed by MALDI-TOF–MS/MS for peptide identification after trypsin digestion. Among these proteins, the nine upregulated proteins were identified as tubulin beta 2C, dynactin subunit 2 (DCTN2), keratin type II cytoskeletal 7, keratin type I cytoskeletal 10, N-myc downstream-regulated gene 1 (NDRG1), heat shock protein beta-1 (HSPB1, Hsp27), phosphatidylinositol transfer protein beta isoform (PITPNB), alpha-enolase isoform 1 (ENO1), and WD repeat-containing protein 1 isoform 1 (WDR1). The 13 downregulated proteins were identified as 14-3-3 gamma (YWHAG), microtubule-associated protein RP/EB family member 1 (MAPRE1), chloride intracellular channel protein 1 (CLIC1), WD-40 repeat protein (STRAP), thioredoxin-like protein (GLRX3), ACTB protein, xaa-Pro dipeptidase isoform 3 (PEPD), enoyl-CoA delta isomerase 1 mitochondrial (ECI1), protein-disulfide isomerase-related chaperone Erp29 (ERP29), ectonucleoside triphosphate diphosphohydrolase 5 isoform (ENTPD5), hnRNP 2H9B (HNRNPH3), peroxiredoxin 1 (PRDX1), and mitotic checkpoint protein BUB3 isoform A (BUB3). The main properties of the identified proteins were summarized in Table 2.

**Table 1:** **Differentially expressed proteins between *C*. *cicadae*-treated and non-treated (control) MHCC97H cells**

**Spot No.**^**1**^	**Protein name**	**GenInfo identifier**^**2**^	**Protein score**^**3**^	**Expression quantity (×10**^**4**^**) control**^**4**^	**Expression quantity (×10**^**4**^**) *C*. *cicadae***	**Expression change ( *C *. *Cicadae*/control)**	***P***	***pI***^**5**^	***Mr *** **(kDa)**^**5**^
0111	14-3-3 Gamma In Complex With A Phosphoserine Peptide	gi|82407948	76	33.0 ± 7.2	19.0 ± 5.3	0.58	0.0004	4.80	28.3
1217	microtubule-associated protein RP/EB family member 1	gi|6912494	219	10.0 ± 3.7	5.7 ± 2.2	0.57	0.026	5.02	30.1
1222	Chloride intracellular channel protein 1	gi|4588526	307	24.8 ± 8.4	9.3 ± 3.3	0.38	0.004112	5.02	27.2
1305	WD-40 repeat protein	gi|4519417	221	42.3 ± 10.2	12.0 ± 4.1	0.28	0.001183	4.93	38.8
1427	Tubulin, beta 2C	gi|23958133	188	3.6 ± 1.7	7.8 ± 4.2	2.17	0.025345	4.83	50.2
1514	Dynactin subunit 2	gi|22096346	436	9.8 ± 2.4	19.2 ± 5.8	1.97	0.000491	5.10	44.3
2303	thioredoxin-like protein	gi|3646128	123	9.3 ± 5.4	3.4 ± 1.9	0.37	0.038567	5.25	37.8
2403	ACTB protein	gi|15277503	444	101.7 ± 33.4	60.3 ± 18.3	0.59	0.001814	5.55	40.5
3413	N-myc downstream regulated gene 1	gi|119612570	105	9.0 ± 3.3	30.8 ± 11.8	3.44	0.00106	5.98	34.0
4102	heat shock protein beta-1	gi|4504517	368	18.1 ± 7.6	27.7 ± 10.6	1.53	0.026641	5.98	*22.8*
4504	xaa-Pro dipeptidase isoform 3	gi|260593665	193	8.7 ± 2.7	3.3 ± 1.2	0.38	0.027093	5.7	48.0
5101	Enoyl-CoA delta isomerase 1, mitochondrial	gi|60593479	67	11.8 ± 7.8	5.8 ± 2.9	0.49	0.040511	6.00	28.9
5106	The Protein-Disulfide Isomerase Related Chaperone Erp29	gi|192987144	247	16.3 ± 6.8	9.3 ± 4.3	0.57	0.033331	7.07	27.2
5218	phosphatidylinositol transfer protein beta isoform	gi|6912594	181	5.5 ± 1.9	8.5 ± 2.6	1.56	0.004165	6.41	31.8
5425	ectonucleoside triphosphate diphosphohydrolase 5, isoform	gi|119601555	181	5.9 ± 2.1	2.3 ± 0.8	0.39	0.007848	5.74	45.7
6334	hnRNP 2H9B	gi|7739445	127	31.1 ± 12.6	17.4 ± 6.5	0.56	0.023	6.76	31.5
6407	alpha-enolase isoform 1	gi|4503571	349	21.4 ± 7.5	34.8 ± 12.6	1.63	0.028265	7.01	47.5
6730	WD repeat-containing protein 1 isoform 1	gi|9257257	217	9.0 ± 4.6	13.9 ± 6.3	1.54	0.01346	6.17	66.8
7122	peroxiredoxin 1	gi|55959887	79	2.7 ± 1.0	0.5 ± 0.1	0.20	0.024662	6.41	19.1
7306	mitotic checkpoint protein BUB3 isoform a	gi|4757880	147	37.1 ± 17.4	22.1 ± 10.1	0.60	0.045568	6.36	37.6

^1^Spot no.: automatically assigned by the PDQuest software.^2^GenInfo identifier: sequence identification number assigned by GenBank.

^3^Protein score: generated by the MS identification system.

^4^Expression Quantity were calculated by the PDQuest software, data expressed as mean ± SD.

^5^*Mr* and *pI*: relative molecular mass (*Mr*) and isoelectric point (*pI*) generated by the MS system.

**Table 2:** The major bio-functions of identified proteins

**Protein name**	**Protein symbol**	**Subcellular location**	**Protein functions**	**Up/down regulation**
**Cell growth and cell cycle regulation**				
14-3-3 Gamma, tyrosine 3-monooxygenase/tryptophan 5-monooxygenase activation protein, gamma	14-3-3γ	Cytoplasm	G2 DNA damage checkpoint [37]	↓
mitotic checkpoint protein BUB3 isoform A	BUB3	Nucleus	Spindle-assembly checkpoint signalling and establishment of correct kinetochore-microtubule attachments [38]	↓
microtubule-associated protein RP/EB family member 1	MAPRE1	Cytoplasm	Microtubule polymerization [39]	↓
Dynactin subunit 2	DCTN2	Cytoplasm	Modulates cytoplasmic dynein binding to an organelle, and prometaphase chromosome alignment and spindle organization during mitosis [40]	↑
thioredoxin-like protein	GLRX3	Cytoplasm	Human cancer cell growth and metastasis regulation [41]	↓
Chloride intracellular channel protein 1	CLIC1	Nucleus membrane, cell membrane	Formation of chloride ion channels, and regulation of the cell cycle [42]	↓
**Anti-cancer effects**				
ectonucleoside triphosphate diphosphohydrolase 5, isoform	ENTPD5	Endoplasmic reticulum lumen	Promoting glycolysis in proliferating cells in response to phosphoinositide 3-kinase signalling in the AKT1-PTEN signalling pathway [43]	↓
N-myc downstream regulated gene 1	NDRG1	Cytoplasm, nucleus, cell membrane	Metastasis suppression [44]	↑
heat shock protein beta-1	HSPB1	Cytoplasm, nucleus	Stress resistance and actin organization [45]	↑
alpha-enolase isoform 1	ENO1	Cytoplasm, cell membrane	Multifunctional enzyme in various processes such as growth control, hypoxia tolerance and allergic responses [46]	↑
**Other functions**				
xaa-Pro dipeptidase isoform 3	PEPD	Cytoplasm	Collagen and GSH metabolism [47]	↓
Enoyl-CoA delta isomerase 1, mitochondrial	ECI1	Mitochondrion matrix		↓
The Protein-Disulfide Isomerase Related Chaperone Erp29	ERP29	Endoplasmic reticulum lumen	Processing of secretory proteins within the endoplasmic reticulum, possibly by participating in the folding of proteins in the ER [48]	↓
phosphatidylinositol transfer protein beta isoform	PITPNB	Cytoplasm, Golgi apparatus	Catalyzes the transfer of PtdIns and phosphatidylcholine between membranes [49]	↑
hnRNP 2H9B	HNRNPH3	Nucleus	Involved in the splicing process and participates in early heat shock-induced splicing arrest [50]	↓
WD repeat-containing protein 1 isoform 1	WDR1	Cytoplasm	Induces disassembly of actin filaments in conjunction with ADF/cofilin family proteins [51]	↑
peroxiredoxin 1	PRDX1	Cytoplasm	Involved in redox regulation of the cell. Reduces peroxides with reducing equivalents provided through the thioredoxin system but not from glutaredoxin [52]	↓
WD-40 repeat protein, serine/threonine kinase receptor associated protein	STRAP	Cytoplasm, nucleus	The SMN complex plays an essential role in spliceosomal snRNP assembly in the cytoplasm and is required for pre-mRNA splicing in the nucleus [53,54]	↓

**Figure 5:**
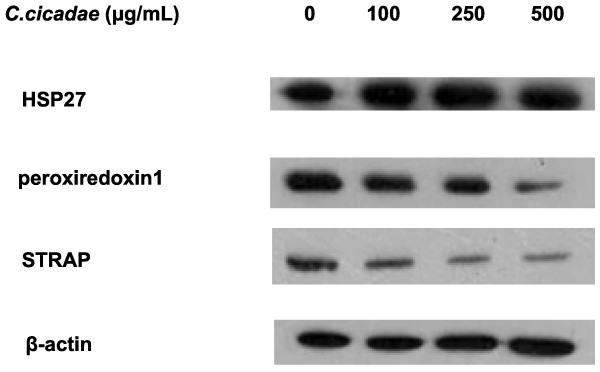
**Western blotting analysis of differential expressed proteins.** With 500 μg/mL of *C. cicadae* treatment, the expression of Hsp27 was upregulated and Peroxiredoxin 1 and WD 40 were up-regulated in the cancer cells as compared to control (500 μg/mL of *C. cicadae*).

Western blot analyses of a few randomly selected proteins were performed for proteomic data validation. In agreement with the proteome changes identified by the proteomic analysis, the expression levels of Hsp27, peroxiredoxin 1, and WD 40 were upregulated in the cancer cells with *C. cicadae* treatment at 500 μg/mL compared with the control cells (Figure 5). These results were similar to those obtained in the proteomic analysis.

## Discussion

Species in the genus *Cordyceps* can reduce cancer cell proliferation [14,15,55-58], but only limited studies on liver cancer are available. Several polysaccharides isolated from *C. cicadae* exhibited antitumor and immunomodulatory properties [14,15,55-58] but the mechanisms have not been fully explored. In the present study, we showed that *C. cicadae* inhibited the growth of the human HCC cell line MHCC97H *via* G2/M cell cycle arrest. In support of the cell cycle measurements, several differentially expressed proteins for cell cycle regulation, mitosis, and protein synthesis were identified by the proteomic analysis.

Western blot analyses revealed a decrease in the level of CDK1, which is a G2/M checkpoint cyclin. The cell cycle measurements also indicated accumulation of cells in G2/M phase rather than induction of apoptosis with *C. cicadae*. To our knowledge, this is the first study to demonstrate that the anticancer mechanisms of *C. cicadae* involve G2/M arrest. Other studies showed that *C. militaris* could induce G2/M cell cycle arrest in human colon cancer HT-29 cells [37].

**Figure 6:**
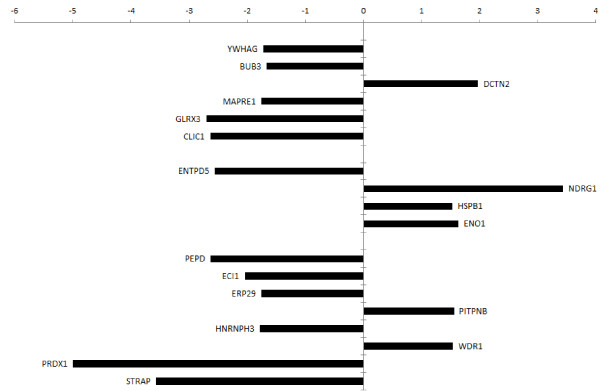
**Summary of the differential expressed proteins in response to *C*. *cicadae *treatment in MHCC97H.** The proteins with increased expression level after 500 μg/mL *C. cicadae* treatment were plotted on the right hand side of axis, and proteins with decreased expression level were plotted on the left hand side.

Figure 6 summarizes the magnitude changes in expression of the identified proteins with reference to the control cells. 14-3-3 gamma, BUB3, DCTN2, MAPRE1, GLRX3, and CLIC1 are thought to be involved in cell cycle regulation, G2/M checkpoint control, microtubule polymerization, and spindle organization during mitosis *etc*. Although their mechanisms related to the G2/M cell cycle arrest induced by *C. cicadae* were not clear, 14-3-3 gamma is important for cell cycle regulation and 14-3-3 gamma expression change-induced cell cycle deregulation has been implicated in cancer formation [37]. 14-3-3 gamma blocked premature mitotic entry into G2/M phase after DNA damage [59,60], while 14-3-3 gamma-knockdown cells showed inhibition of cdc25C function, which induced overriding of both the incomplete S phase and the G2 DNA damage checkpoint [61]. Overexpression of 14-3-3 gamma in HCC patients was associated with extrahepatic metastasis and worse survival [62,63]. Our proteomic data showed that *C. cicadae* treatment reduced the expression of 14-3-3 gamma in the human HCC cell line MHCC97H, suggesting that the anticancer and G2/M phase arrest effects of *C. cicadae* could be attributed to 14-3-3 gamma expression inhibition.

BUB3, DCTN2, and MAPRE1 are involved in spindle checkpoint and mitosis regulation. BUB3 is a mitosis checkpoint protein for early stages of mitosis to prevent premature sister chromatid separation, missegregation, and aneuploidy *via* regulation of anaphase-promoting complex/cyclosome (APC/C) [38,64]. DCTN2 is involved in functions for the microtubule anchor dynactin complex, inactivation of the spindle checkpoint through kinetochore disassembly, and recruitment of cell cycle regulators to centrosomes [40,65]. MAPRE1 is involved in microtubule polymerization, microtubule dynamics control [39], and mitotic spindle function [66], and is dominantly expressed in poorly differentiated HCCs [67]. The expression changes of BUB3, DCTN2, and MAPRE1 in *C. cicadae*-treated MHCC97H cells indicate a mitosis-regulatory effect of *C. cicadae*, which may contribute to G2/M phase arrest.

GLRX3 maintains redox homeostasis in living cells [41]. In GLRX3-knockout mice, embryonic fibroblasts were arrested at G2/M phase [68]. CLIC1 is a nuclear chloride ion channel and involved in G2/M cell cycle regulation [42]. These findings may suggest a reason why the expression levels of GLRX3 and CLIC1 were changed in *C. cicadae*-treated MHCC97H cells.

ENTPD5 is an endoplasmic reticulum (ER) enzyme that hydrolyzes purine nucleoside diphosphates and is essential for liver functions, and ENTPD5-deficiency resulted in hepatocellular neoplasia [69]. Overexpression of ENTPD5 was observed in prostate cancer [43,70]. NDRG1 is a member of the NDRG gene family, and exhibited anticancer and metastasis-suppression effects in pancreatic cancer, colon cancer, cervical and ovarian cancer, prostate cancer, and breast cancer [44,71-73]. MHCC97H cells exhibited an 80% pulmonary metastatic rate when injected into nude mice [74]. HSPB1 is a subunit of HSP27, and suppressed cell apoptosis in MHCC97H cells *via* an NF-kappaB pathway [45]. The elevation of this protein indicated some resistance of the cancer cells, and could be responsible for the absence of apoptosis in the *C. cicadae*-treated cancer cells. ENO1, a glycolysis module protein, was overexpressed in HCC cells [46,75], and its increased level suggests that *C. cicadae* treatment increased the glycolytic activity of the cancer cells.

A reduction in PEPD is involved in collagen and GSH metabolism of *C. cicadae*. PEPD is a homodimeric iminodipeptidase that releases carboxy-terminal proline or hydroxyproline residues from oligopeptides, and is used as a marker of non-alcoholic liver fibrosis [47,76]. ERP29 works in the early secretory pathway in the ER, and overexpression of ERP29 resulted in higher expression of HSP27 in breast cancer cells [48]. The reduced expression of ERP29 is likely to be attributed to reduced ER stress with treatment of *C. cicadae*.

STRAP is involved in spliceosomal mRNP assembly in the cytoplasm and pre-mRNA splicing in the nucleus [53]. Overexpression of STRAP was reported in several cancers [53,77]. A STRAP-activated p53-related apoptosis-induction effect and higher expression of STRAP in drug-treated HCC cells were observed [54,78]. In the present study, the expression of STRAP was reduced, which may be related to the non-apoptotic effect of *C. cicadae*.

PRDX1 is a member of the peroxiredoxin family involved in redox regulation in cells, regulation of hydrogen peroxide signaling through its peroxidase activity, and a protein chaperone function. The aspect of whether the reduced expression of PRDX1 contributes to reduced survival of MHCC97H cells *via* failure in redox regulation against reactive oxygen species remains to be determined. Elevated expression of PRDX1 was found in various cancers [52,79,80].

The present study has identified novel molecular signatures in *C. cicadae*-induced cell cycle arrest and cell death in MHCC97H cells using proteomics. Among the differentially expressed proteins identified, 14-3-3 gamma, BUB3, DCTN2, MAPRE1, GLRX3, and CLIC1 may play some significant roles in the *C. cicadae*-induced G2/M phase arrest in MHCC97H cells. The identified proteins will further enhance a molecular explanation for the cell cycle arrest process induced by *C. cicadae*. Further validation of these markers in gene knockout studies would greatly improve our understanding of the molecular mechanisms behind the liver cancer therapy potential of *C. cicadae*.

## Conclusions

The water extract of *C. cicadae* reduced the growth of the human HCC cell line MHCC97H *via* G2/M cell cycle arrest.

## Competing interests

The authors declare that they have no competing interests.

## Authors’ contributions

HW and JMFW conceived and designed the study. HLW, ZJ and WHS performed experiments. HLW performed data analysis. HW, CYJL and JMFW wrote the manuscript. All authors read and approved the final version of the manuscript.

## References

[B1] Ferlay JSH Bray F Forman D Mathers C Parkin DM Cancer Incidence and Mortality Worldwide: IARC CancerBase No. 10 2008 http://globocan.iarc.fr/Default.aspx

[B2] Shiu WC Primary liver cancer in Hong Kong Cancer Chemother Pharmacol 1992 31 Suppl S143 S145 1458565 10.1007/BF00687124

[B3] Chen JG Zhang SW Liver cancer epidemic in China: past, present and future Semin Cancer Biol 2011 21 59 69 10.1016/j.semcancer.2010.11.002 21144900

[B4] Chao J Chang ET So SK Hepatitis B and liver cancer knowledge and practices among healthcare and public health professionals in China: a cross-sectional study BMC Public Health 2010 10 98 10.1186/1471-2458-10-98 20184740 PMC2847983

[B5] Wu P Dugoua JJ Eyawo O Mills EJ Traditional Chinese Medicines in the treatment of hepatocellular cancers: a systematic review and meta-analysis J Exp Clin Cancer Res 2009 28 112 10.1186/1756-9966-28-112 19674474 PMC3225807

[B6] Winkler D *Yartsa Gunbu* (*Cordyceps sinensis*) and the Fungal Commodification of Tibet’s Rural Economy Econ Bot 2008 62 291 305 10.1007/s12231-008-9038-3

[B7] Li FH Liu P Xiong WG Xu GF [Effects of Cordyceps sinensis on dimethylnitrosamine-induced liver fibrosis in rats] Zhong Xi Yi Jie He Xue Bao 2006 4 514 517 16965748 10.3736/jcim20060515

[B8] Wu JY Zhang QX Leung PH Inhibitory effects of ethyl acetate extract of Cordyceps sinensis mycelium on various cancer cells in culture and B16 melanoma in C57BL/6 mice Phytomedicine 2007 14 43 49 16423520 10.1016/j.phymed.2005.11.005

[B9] Zhang Q Wu J Hu Z Li D Induction of HL-60 apoptosis by ethyl acetate extract of Cordyceps sinensis fungal mycelium Life Sci 2004 75 2911 2919 10.1016/j.lfs.2004.05.029 15454342

[B10] Yang HY Leu SF Wang YK Wu CS Huang BM Cordyceps sinensis mycelium induces MA-10 mouse Leydig tumor cell apoptosis by activating the caspase-8 pathway and suppressing the NF-kappaB pathway Arch Androl 2006 52 103 110 10.1080/01485010500315818 16443586

[B11] Nakamura K Konoha K Yamaguchi Y Kagota S Shinozuka K Kunitomo M Combined effects of Cordyceps sinensis and methotrexate on hematogenic lung metastasis in mice Receptors Channels 2003 9 329 334 14527877 10.3109/713745176

[B12] Kubo E Yoshikawa N Kunitomo M Kagota S Shinozuka K Nakamura K Inhibitory effect of Cordyceps sinensis on experimental hepatic metastasis of melanoma by suppressing tumor cell invasion Anticancer Res 2010 30 3429 3433 20944118

[B13] Park C Hong SH Lee JY Kim GY Choi BT Lee YT Park DI Park YM Jeong YK Choi YH Growth inhibition of U937 leukemia cells by aqueous extract of Cordyceps militaris through induction of apoptosis Oncol Rep 2005 13 1211 1216 15870944

[B14] Lee H Kim YJ Kim HW Lee DH Sung MK Park T Induction of apoptosis by Cordyceps militaris through activation of caspase-3 in leukemia HL-60 cells Biol Pharm Bull 2006 29 670 674 10.1248/bpb.29.670 16595897

[B15] Park SE Yoo HS Jin CY Hong SH Lee YW Kim BW Lee SH Kim WJ Cho CK Choi YH Induction of apoptosis and inhibition of telomerase activity in human lung carcinoma cells by the water extract of Cordyceps militaris Food Chem Toxicol 2009 47 1667 1675 10.1016/j.fct.2009.04.014 19393284

[B16] Park SE Kim J Lee YW Yoo HS Cho CK Antitumor activity of water extracts from Cordyceps militaris in NCI-H460 cell xenografted nude mice J Acupunct Meridian Stud 2009 2 294 300 10.1016/S2005-2901(09)60071-6 20633505

[B17] Jin CY Kim GY Choi YH Induction of apoptosis by aqueous extract of Cordyceps militaris through activation of caspases and inactivation of Akt in human breast cancer MDA-MB-231 Cells J Microbiol Biotechnol 2008 18 1997 2003 19131705

[B18] Lee SJ Kim SK Choi WS Kim WJ Moon SK Cordycepin causes p21WAF1-mediated G2/M cell-cycle arrest by regulating c-Jun N-terminal kinase activation in human bladder cancer cells Arch Biochem Biophys 2009 490 103 109 10.1016/j.abb.2009.09.001 19733546

[B19] Yoshikawa N Kunitomo M Kagota S Shinozuka K Nakamura K Inhibitory effect of cordycepin on hematogenic metastasis of B16-F1 mouse melanoma cells accelerated by adenosine-5’-diphosphate Anticancer Res 2009 29 3857 3860 19846919

[B20] Wu WC Hsiao JR Lian YY Lin CY Huang BM The apoptotic effect of cordycepin on human OEC-M1 oral cancer cell line Cancer Chemother Pharmacol 2007 60 103 111 10.1007/s00280-006-0354-y 17031645

[B21] Choi S Lim MH Kim KM Jeon BH Song WO Kim TW Cordycepin-induced apoptosis and autophagy in breast cancer cells are independent of the estrogen receptor Toxicol Appl Pharmacol 2011 257 165 173 10.1016/j.taap.2011.08.030 21933677

[B22] Jeong JW Jin CY Park C Hong SH Kim GY Jeong YK Lee JD Yoo YH Choi YH Induction of apoptosis by cordycepin via reactive oxygen species generation in human leukemia cells Toxicol In Vitro 2011 25 817 824 10.1016/j.tiv.2011.02.001 21310227

[B23] Ukai S Kiho T Hara C Morita M Goto A Imaizumi N Hasegawa Y Polysaccharides in fungi. XIII. Antitumor activity of various polysaccharides isolated from Dictyophora indusiata, Ganoderma japonicum, Cordyceps cicadae, Auricularia auricula-judae, and Auricularia species Chem Pharm Bull (Tokyo) 1983 31 741 744 10.1248/cpb.31.741 6883594

[B24] Yang JZ Zhuo J Chen BK Jin LQ Lv JX Li LJ [Regulating effects of Paecilomyces cicadae polysaccharides on immunity of aged rats] Zhongguo Zhong Yao Za Zhi 2008 33 292 295 18536469

[B25] Zhu R Chen YP Deng YY Zheng R Zhong YF Wang L Du LP Cordyceps cicadae extracts ameliorate renal malfunction in a remnant kidney model J Zhejiang Univ Sci B 2011 12 1024 1033 10.1631/jzus.B1100034 22135152 PMC3232436

[B26] Weng SC Chou CJ Lin LC Tsai WJ Kuo YC Immunomodulatory functions of extracts from the Chinese medicinal fungus Cordyceps cicadae J Ethnopharmacol 2002 83 79 85 10.1016/S0378-8741(02)00212-X 12413710

[B27] Kim HS Kim JY Ryu HS Shin BR Kang JS Kim HM Kim YO Hong JT Kim Y Han SB Phenotypic and functional maturation of dendritic cells induced by polysaccharide isolated from Paecilomyces cicadae J Med Food 2011 14 847 856 10.1089/jmf.2011.1575 21631358

[B28] Kuo YC Weng SC Chou CJ Chang TT Tsai WJ Activation and proliferation signals in primary human T lymphocytes inhibited by ergosterol peroxide isolated from Cordyceps cicadae Br J Pharmacol 2003 140 895 906 10.1038/sj.bjp.0705500 14504132 PMC1574094

[B29] Zhu R Zheng R Deng Y Chen Y Zhang S Ergosterol peroxide from Cordyceps cicadae ameliorates TGF-beta1-induced activation of kidney fibroblasts Phytomedicine 2013 21 3 372 378 24095053 10.1016/j.phymed.2013.08.022

[B30] Ju-fen JZ-m CAI Hong-yang LU Bo-zheng LU Research of Anti - tumor Effects of Different Purified Components of Cordyceps Cicadae in Vitro Chin Arch Tradit Chin Med 2010 28 5

[B31] Jin L-Q Xiong Z-K Lu J-X Experimental studies on immunomodulatory and antitumor activity of polysaccharide from Paecilomyces cicadidae Chinese Journal of Pathophysiology 2008 24 4

[B32] Hondermarck H Proteomics: Biomedical and Pharmaceutical Applications 2004 Dordrecht: Kluwer Academic Publishers

[B33] Rajcevic U Niclou SP Jimenez CR Proteomics strategies for target identification and biomarker discovery in cancer Front Biosci 2009 14 3292 3303 10.2741/3452 19273274

[B34] Lee CY Sit WH Fan ST Man K Jor IW Wong LL Wan ML Tan-Un KC Wan JM The cell cycle effects of docosahexaenoic acid on human metastatic hepatocellular carcinoma proliferation Int J Oncol 2010 36 991 998 20198345 10.3892/ijo_00000579

[B35] Jiang P Siggers JL Ngai HH Sit WH Sangild PT Wan JM The small intestine proteome is changed in preterm pigs developing necrotizing enterocolitis in response to formula feeding J Nutr 2008 138 1895 1901 18806098 10.1093/jn/138.10.1895

[B36] Ferrell JE Jr Wu M Gerhart JC Martin GS Cell cycle tyrosine phosphorylation of p34cdc2 and a microtubule-associated protein kinase homolog in Xenopus oocytes and eggs Mol Cell Biol 1991 11 1965 1971 2005892 10.1128/mcb.11.4.1965-1971.1991 PMC359881

[B37] Hermeking H Benzinger A 14-3-3 proteins in cell cycle regulation Semin Cancer Biol 2006 16 183 192 10.1016/j.semcancer.2006.03.002 16697662

[B38] Lopes CS Sampaio P Williams B Goldberg M Sunkel CE The Drosophila Bub3 protein is required for the mitotic checkpoint and for normal accumulation of cyclins during G2 and early stages of mitosis J Cell Sci 2005 118 187 198 10.1242/jcs.01602 15615783

[B39] Honnappa S Gouveia SM Weisbrich A Damberger FF Bhavesh NS Jawhari H Grigoriev I van Rijssel FJA Buey RM Lawera A Jelesarov I Winkler FK Wüthrich K Akhmanova A Steinmetz MO An EB1-Binding Motif Acts as a Microtubule Tip Localization Signal Cell 2009 138 366 376 10.1016/j.cell.2009.04.065 19632184

[B40] Quintyne NJ Schroer TA Distinct cell cycle–dependent roles for dynactin and dynein at centrosomes J Cell Biol 2002 159 245 254 10.1083/jcb.200203089 12391026 PMC2173046

[B41] Haunhorst P Hanschmann EM Brautigam L Stehling O Hoffmann B Muhlenhoff U Lill R Berndt C Lillig CH Crucial function of vertebrate glutaredoxin 3 (PICOT) in iron homeostasis and hemoglobin maturation Mol Biol Cell 2013 24 1895 1903 10.1091/mbc.E12-09-0648 23615448 PMC3681695

[B42] Valenzuela SM Mazzanti M Tonini R Qiu MR Warton K Musgrove EA Campbell TJ Breit SN The nuclear chloride ion channel NCC27 is involved in regulation of the cell cycle J Physiol 2000 529 Pt 3 541 552 11195932 10.1111/j.1469-7793.2000.00541.x PMC2270212

[B43] Villar J Quadri HS Song I Tomita Y Tirado OM Notario V PCPH/ENTPD5 Expression Confers to Prostate Cancer Cells Resistance against Cisplatin-Induced Apoptosis through Protein Kinase Cα–Mediated Bcl-2 Stabilization Cancer Res 2009 69 102 110 19117992 10.1158/0008-5472.CAN-08-2922 PMC2614304

[B44] Angst E Dawson DW Stroka D Gloor B Park J Candinas D Reber HA Hines OJ Eibl G N-myc downstream regulated gene-1 expression correlates with reduced pancreatic cancer growth and increased apoptosis in vitro and in vivo Surgery 2011 149 614 624 10.1016/j.surg.2010.11.002 21236457

[B45] Guo K Kang NX Li Y Sun L Gan L Cui FJ Gao MD Liu KY Regulation of HSP27 on NF-kappaB pathway activation may be involved in metastatic hepatocellular carcinoma cells apoptosis BMC Cancer 2009 9 100 10.1186/1471-2407-9-100 19331697 PMC2681475

[B46] Hamaguchi T Iizuka N Tsunedomi R Hamamoto Y Miyamoto T Iida M Tokuhisa Y Sakamoto K Takashima M Tamesa T Oka M Glycolysis module activated by hypoxia-inducible factor 1alpha is related to the aggressive phenotype of hepatocellular carcinoma Int J Oncol 2008 33 725 731 18813785

[B47] Horoz M Aslan M Bolukbas FF Bolukbas C Nazligul Y Celik H Aksoy N Serum prolidase enzyme activity and its relation to histopathological findings in patients with non-alcoholic steatohepatitis J Clin Lab Anal 2010 24 207 211 10.1002/jcla.20334 20486204 PMC6647739

[B48] Zhang D Putti TC Over-expression of ERp29 attenuates doxorubicin-induced cell apoptosis through up-regulation of Hsp27 in breast cancer cells Exp Cell Res 2010 316 3522 3531 10.1016/j.yexcr.2010.08.014 20833165

[B49] Carvou N Holic R Li M Futter C Skippen A Cockcroft S Phosphatidylinositol- and phosphatidylcholine-transfer activity of PITPbeta is essential for COPI-mediated retrograde transport from the Golgi to the endoplasmic reticulum J Cell Sci 2010 123 1262 1273 10.1242/jcs.061986 20332109 PMC2848114

[B50] Garneau D Revil T Fisette J-F Chabot B Heterogeneous Nuclear Ribonucleoprotein F/H Proteins Modulate the Alternative Splicing of the Apoptotic Mediator Bcl-x J Biol Chem 2005 280 22641 22650 10.1074/jbc.M501070200 15837790

[B51] Fujibuchi T Abe Y Takeuchi T Imai Y Kamei Y Murase R Ueda N Shigemoto K Yamamoto H Kito K AIP1/WDR1 supports mitotic cell rounding Biochem Biophys Res Commun 2005 327 268 275 10.1016/j.bbrc.2004.11.156 15629458

[B52] Noh DY Ahn SJ Lee RA Kim SW Park IA Chae HZ Overexpression of peroxiredoxin in human breast cancer Anticancer Res 2001 21 2085 2090 11497302

[B53] Halder SK Anumanthan G Maddula R Mann J Chytil A Gonzalez AL Washington MK Moses HL Beauchamp RD Datta PK Oncogenic function of a novel WD-domain protein, STRAP, in human carcinogenesis Cancer Res 2006 66 6156 6166 10.1158/0008-5472.CAN-05-3261 16778189

[B54] Jung H Seong HA Ha H NM23-H1 tumor suppressor and its interacting partner STRAP activate p53 function J Biol Chem 2007 282 35293 35307 10.1074/jbc.M705181200 17916563

[B55] Jiang J Sliva D Novel medicinal mushroom blend suppresses growth and invasiveness of human breast cancer cells Int J Oncol 2010 37 1529 1536 21042722 10.3892/ijo_00000806

[B56] Marchbank T Ojobo E Playford CJ Playford RJ Reparative properties of the traditional Chinese medicine Cordyceps sinensis (Chinese caterpillar mushroom) using HT29 cell culture and rat gastric damage models of injury Br J Nutr 2011 105 1303 1310 10.1017/S0007114510005118 21272405

[B57] Kim HG Song H Yoon DH Song BW Park SM Sung GH Cho JY Park HI Choi S Song WO Hwang KC Kim TW Cordyceps pruinosa extracts induce apoptosis of HeLa cells by a caspase dependent pathway J Ethnopharmacol 2010 128 342 351 10.1016/j.jep.2010.01.049 20138133

[B58] Oh JY Baek YM Kim SW Hwang HJ Hwang HS Lee SH Yun JW Apoptosis of human hepatocarcinoma (HepG2) and neuroblastoma (SKN-SH) cells induced by polysaccharides-peptide complexes produced by submerged mycelial culture of an entomopathogenic fungus Cordyceps sphecocephala J Microbiol Biotechnol 2008 18 512 519 18388470

[B59] Dalal SN Yaffe MB DeCaprio JA 14-3-3 family members act coordinately to regulate mitotic progression Cell Cycle 2004 3 672 677 15107609

[B60] Kasahara K Goto H Enomoto M Tomono Y Kiyono T Inagaki M 14-3-3gamma mediates Cdc25A proteolysis to block premature mitotic entry after DNA damage EMBO J 2010 29 2802 2812 10.1038/emboj.2010.157 20639859 PMC2924644

[B61] Hosing AS Kundu ST Dalal SN 14-3-3 Gamma is required to enforce both the incomplete S phase and G2 DNA damage checkpoints Cell Cycle 2008 7 3171 3179 10.4161/cc.7.20.6812 18843201

[B62] Lee IN Chen CH Sheu JC Lee HS Huang GT Yu CY Lu FJ Chow LP Identification of human hepatocellular carcinoma-related biomarkers by two-dimensional difference gel electrophoresis and mass spectrometry J Proteome Res 2005 4 2062 2069 10.1021/pr0502018 16335951

[B63] Ko BS Lai IR Chang TC Liu TA Chen SC Wang J Jan YJ Liou JY Involvement of 14-3-3gamma overexpression in extrahepatic metastasis of hepatocellular carcinoma Hum Pathol 2011 42 129 135 10.1016/j.humpath.2010.01.028 20870266

[B64] Logarinho E Resende T Torres C Bousbaa H The human spindle assembly checkpoint protein Bub3 is required for the establishment of efficient kinetochore-microtubule attachments Mol Biol Cell 2008 19 1798 1813 10.1091/mbc.E07-07-0633 18199686 PMC2291436

[B65] Howell BJ McEwen BF Canman JC Hoffman DB Farrar EM Rieder CL Salmon ED Cytoplasmic dynein/dynactin drives kinetochore protein transport to the spindle poles and has a role in mitotic spindle checkpoint inactivation J Cell Biol 2001 155 1159 1172 10.1083/jcb.200105093 11756470 PMC2199338

[B66] Green RA Wollman R Kaplan KB APC and EB1 function together in mitosis to regulate spindle dynamics and chromosome alignment Mol Biol Cell 2005 16 4609 4622 10.1091/mbc.E05-03-0259 16030254 PMC1237068

[B67] Orimo T Ojima H Hiraoka N Saito S Kosuge T Kakisaka T Yokoo H Nakanishi K Kamiyama T Todo S Hirohashi S Kondo T Proteomic profiling reveals the prognostic value of adenomatous polyposis coli-end-binding protein 1 in hepatocellular carcinoma Hepatology 2008 48 1851 1863 10.1002/hep.22552 18937283

[B68] Cheng NH Zhang W Chen WQ Jin J Cui X Butte NF Chan L Hirschi KD A mammalian monothiol glutaredoxin, Grx3, is critical for cell cycle progression during embryogenesis FEBS J 2011 278 2525 2539 21575136 10.1111/j.1742-4658.2011.08178.x PMC4038268

[B69] Read R Hansen G Kramer J Finch R Li L Vogel P Ectonucleoside triphosphate diphosphohydrolase type 5 (Entpd5)-deficient mice develop progressive hepatopathy, hepatocellular tumors, and spermatogenic arrest Vet Pathol 2009 46 491 504 10.1354/vp.08-VP-0201-R-AM 19176496

[B70] Villar J Arenas MI MacCarthy CM Blanquez MJ Tirado OM Notario V PCPH/ENTPD5 expression enhances the invasiveness of human prostate cancer cells by a protein kinase C delta-dependent mechanism Cancer Res 2007 67 10859 10868 10.1158/0008-5472.CAN-07-2041 18006831

[B71] Bandyopadhyay S Pai SK Hirota S Hosobe S Tsukada T Miura K Takano Y Saito K Commes T Piquemal D Watabe M Gross S Wang Y Huggenvik J Watabe K PTEN up-regulates the tumor metastasis suppressor gene Drg-1 in prostate and breast cancer Cancer Res 2004 64 7655 7660 10.1158/0008-5472.CAN-04-1623 15520163

[B72] Li Q Chen H Transcriptional silencing of N-Myc downstream-regulated gene 1 (NDRG1) in metastatic colon cancer cell line SW620 Clin Exp Metastasis 2011 28 127 135 10.1007/s10585-010-9366-4 21184144

[B73] Zhao G Chen J Deng Y Gao F Zhu J Feng Z Lv X Zhao Z Identification of NDRG1-regulated genes associated with invasive potential in cervical and ovarian cancer cells Biochem Biophys Res Commun 2011 408 154 159 10.1016/j.bbrc.2011.03.140 21463610

[B74] Li YL Tang ZT Ye SY Liu BL Liu YL Chen JC Xue QX Establishment of a hepatocellular carcinoma cell line with unique metastatic characteristics through in vivo selection and screening for metastasis-related genes through cDNA microarray J Cancer Res Clin Oncol 2003 129 43 51 12618900 10.1007/s00432-002-0396-4 PMC12161897

[B75] Takashima M Kuramitsu Y Yokoyama Y Iizuka N Fujimoto M Nishisaka T Okita K Oka M Nakamura K Overexpression of alpha enolase in hepatitis C virus-related hepatocellular carcinoma: association with tumor progression as determined by proteomic analysis Proteomics 2005 5 1686 1692 10.1002/pmic.200401022 15800975

[B76] Kayadibi H Gultepe M Yasar B Ince AT Ozcan O Ipcioglu OM Kurdas OO Bolat B Benek YZ Guveli H Atalay S Ozkara S Keskin O Diagnostic value of serum prolidase enzyme activity to predict the liver histological lesions in non-alcoholic fatty liver disease: a surrogate marker to distinguish steatohepatitis from simple steatosis Dig Dis Sci 2009 54 1764 1771 10.1007/s10620-008-0535-0 18989777

[B77] Matsuda S Katsumata R Okuda T Yamamoto T Miyazaki K Senga T Machida K Thant AA Nakatsugawa S Hamaguchi M Molecular cloning and characterization of human MAWD, a novel protein containing WD-40 repeats frequently overexpressed in breast cancer Cancer Res 2000 60 13 17 10646843

[B78] Wang H Ye Y Pan SY Zhu GY Li YW Fong DW Yu ZL Proteomic identification of proteins involved in the anticancer activities of oridonin in HepG2 cells Phytomedicine 2011 18 163 169 10.1016/j.phymed.2010.06.011 20724128

[B79] Qi Y Chiu JF Wang L Kwong DL He QY Comparative proteomic analysis of esophageal squamous cell carcinoma Proteomics 2005 5 2960 2971 10.1002/pmic.200401175 15986332

[B80] Shen J Person MD Zhu J Abbruzzese JL Li D Protein expression profiles in pancreatic adenocarcinoma compared with normal pancreatic tissue and tissue affected by pancreatitis as detected by two-dimensional gel electrophoresis and mass spectrometry Cancer Res 2004 64 9018 9026 10.1158/0008-5472.CAN-04-3262 15604267

